# Facile molten salt synthesis of Cs–MnO_2_ hollow microflowers for supercapacitor applications†

**DOI:** 10.1039/c9ra02067e

**Published:** 2019-06-17

**Authors:** Praeploy Chomkhuntod, Arreerat Jiamprasertboon, Anurak Waehayee, Teera Butburee, Narong Chanlek, Nararat Yong, Theeranun Siritanon

**Affiliations:** School of Chemistry, Institute of Science, Suranaree University of Technology Nakhon Ratchasima 30000 Thailand theeranun@sut.ac.th; National Nanotechnology Center, National Science and Technology Development Agency 111 Thailand Science Park Pathum Thani 12120 Thailand; Synchrotron Light Research Institute Nakhon Ratchasima 30000 Thailand; Center of Excellent-Advanced Functional Materials, Suranaree University of Technology Nakhon Ratchasima 30000 Thailand

## Abstract

A facile molten salt technique is an interesting preparation method as it enables mass production of materials. With the use of CsNO_3_ salt, Cs-intercalated MnO_2_ hollow microflowers are obtained in this work. δ-MnO_2_ with a layered structure, instead of other allotropes with smaller structural cavities, is formed and stabilized by large Cs^+^ ions. Formation of the hollow microflowers is explained based on the Ostwald ripening process. The salt to starting agent ratio has little effect on the crystal structure and morphologies of the products but does influence the crystallinity, the interlayer distance, and the intercalating Cs^+^ content. The capacity of Cs^+^ in the structure and the interlayer distance are maximized when the weight ratio of CsNO_3_ : MnSO_4_ is 7 : 1. Cs–MnO_2_ obtained from this optimum ratio has most suitable crystallinity and interlayer distance, and consequently shows a highest specific capacitance of 155 F g^−1^ with excellent cycling performance. The obtained specific capacitance is comparable to that of other alkaline-intercalated MnO_2_, suggesting that Cs–MnO_2_ could be another interesting candidate for supercapacitor electrodes.

## Abstract 2

Hollow microflowers of Cs intercalated MnO_2_ binessite are prepared by a facile molten salt method and tested for supercapacitor electrodes.

## Graphical abstract

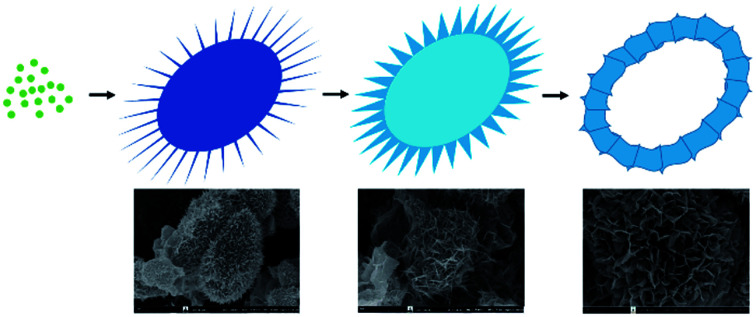

## 1. Introduction

Manganese oxide (MnO_2_) has become an attractive candidate to be used as a promising material for supercapacitor electrodes because of its high performance and low price. It is known that the specific capacitance of MnO_2_ is dependent upon the crystal structure. Among all MnO_2_ allotropes, δ-MnO_2_ (birnessite) with a layered structure is one of the most interesting candidates as it exhibits a high specific capacitance.^1–3^ The 2D structure of δ-MnO_2_ is advantageous for fast ion diffusion into the bulk, which is a preferred characteristic of the electrodes.^3,4^ Besides the crystal structure, morphology is also an important factor. Thus, various preparation techniques have been explored, and some of which interestingly give MnO_2_ with fascinating morphologies and good electrochemical properties.^5–8^ Nevertheless, in addition to giving materials with high specific capacitance, a good preparation method should be simple, low-cost, and easy to be scaled up.

One of the interesting preparing methods is a simple molten salt technique. The molten salt technique employing alkaline nitrates (LiNO_3_, NaNO_3_, and KNO_3_)^9^ and chlorides (KCl, LiCl, and NaCl)^10^ was reported to yield α/β-MnO_2_ and α/γ-MnO_2_ nanowires, respectively. It was postulated that an ionic radius of cationic components of the salts determines a crystal structure of the products. Nitrate salts of smaller ions like Li^+^ and Na^+^ tend to induce the formation of β-MnO_2_, while the salts containing lager ions such as K^+^ promote the formation of α-MnO_2_.^9^ On the other hand, a recent work reported that Na-, K-, and Cs-intercalated δ-MnO_2_ with 2D morphology were obtained *via* a 1 minute molten salt synthesis using NaNO_3_, KNO_3_, and CsNO_3_, respectively.^11^ The inconsistencies in both morphology and crystal structure of MnO_2_ prepared by a similar molten salt method strongly suggest that the preparing conditions including temperature, types of salt, and salt content play important roles. The reaction time is specifically an important factor as it is known that α and β-MnO_2_ are more thermodynamically stable than δ-phase, and the δ-MnO_2_ can transform to other structures with the prolonged reaction time.^12^ Additionally, Li-, Na-, and K-intercalated MnO_2_ birnessite have been studied as candidates for supercapacitor electrodes.^13–16^ Thus, Cs–MnO_2_ could be another interesting candidate for the same applications. Yet, reports on electrochemical behaviors of Cs–MnO_2_ birnessite are scarce.

Here, we aim to study the preparation of MnO_2_ by a molten salt technique using CsNO_3_. Interestingly, we found that CsNO_3_ induces δ-MnO_2_ formation and stabilizes the structure. Moreover, the reagent ratio significantly affects electrochemical properties of the obtained products. As-prepared Cs–MnO_2_ exhibits excellent capacitive behaviors because the intercalating Cs^+^ ion expands the interlayer distance, which could improve ion diffusion during the charge/discharge process.^17^

## 2. Experimental section

### 2.1. Material synthesis

All samples were prepared by molten salt technique using CsNO_3_ salt. In the first part, which aims to study the formation process of the compound, 1.0 g of CsNO_3_ and 0.1 g of MnSO_4_ (10 : 1 weight ratio) were mixed and heated at 430 °C with a ramp rate of 300 °C h in a muffle furnace for the designated time. After naturally cooled to room temperature, the resultant products were washed and dried at 90 °C for 12 h. To study the effect of CsNO_3_ salt to MnSO_4_ weight ratio, similar experiments with varied salt to reagent weight ratio of 3 : 1, 5 : 1, 7 : 1, and 10 : 1 were additionally conducted by keeping mass of MnSO_4_ constant at 0.1 g and the mixtures were heated at 430 °C for 3 hours.

### 2.2. Material characterizations

Phase of the obtained powders were characterized by powder X-ray diffractometer (XRD: D2 Phaser, Bruker with Cu Kα). To calculate the change in the interlayer distance, powder X-ray diffraction patterns of the samples were additionally collected with an addition of KCl as an internal standard. The patterns were then used to calculate interlayer distances by Le Bail method using a TOPAS software. Scanning electron microscope (SEM: FEI quanta 450) and transmission electron microscope (TEM: JEOL2100plus, operated at 200 keV) were used to investigate the sample morphology, composition, and elemental distribution. The Cs : Mn mole ratio was determined by Inductively coupled plasma-optical emission spectrometry (ICP-OES). BELSORP-mini II surface area and pore size analyzer, Bel-Japan, was used to study the Brunauer–Emmett–Teller (BET) surface areas. The chemical composition of the prepared samples was confirmed by X-ray photoelectron spectroscopy (XPS: PHI5000 VersaProbe II, ULVAC-PHI) with a monochromatic Al Kα excitation source (1486.6 eV).

### 2.3. Electrochemical measurements

To prepare the electrode, MnO_2_ samples, carbon black, and polyvinylidene fluoride with 8 : 1 : 1 weight ratio were mixed by grinding in 1-methyl-2-pyrrolidinone to form a homogeneous suspension. The suspension was dropped on a Ni foam until a loading mass of 1 mg was achieved. After drying, the electrodes were characterized by cyclic voltammetry (CV) and galvanostatic charge discharge (GCD) by AUTOLAB instrument electrochemical workstation. The standard three-electrode configuration consisting of Ag/AgCl, platinum, and MnO_2_-coated Ni foam as a reference, counter, and working electrodes; respectively. 0.5 M K_2_SO_4_ was used as an electrolyte in this work.

## 3. Results and discussion

### 3.1. Sample preparation

**Fig. 1:**
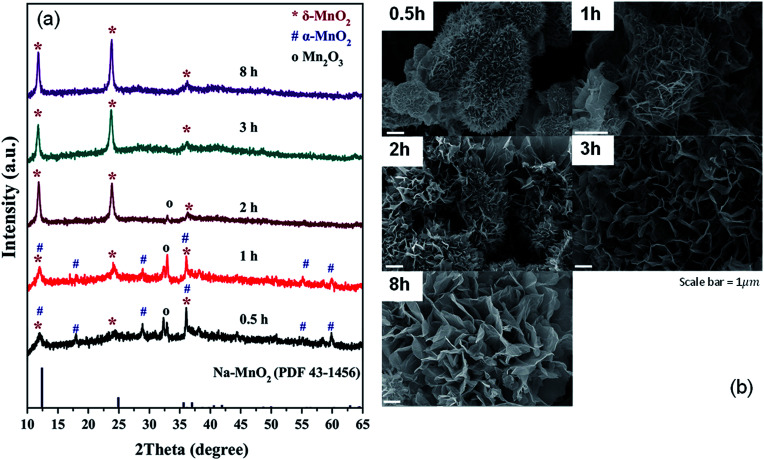
XRD patterns (a) and SEM images (b) of the products from molten salt synthesis with various reaction times. CsNO_3_ : MnSO_4_ weight ratio of 10 : 1 was used.

Based on the XRD patterns (Fig. 1a), a single phase of δ-MnO_2_ is obtained with an appropriate reaction time. The reaction occurs through Mn_2_O_3_ formation as the phase is observed in the samples with 0.5, 1, and 2 hour reaction time. Additionally, it can be deduced that α-MnO_2_ was formed first and later transformed to δ-MnO_2_, which remained the only phase even at 8 hours of reaction. While molten salt reactions using KNO_3_ gave a stable α-MnO_2_,^9^ a large Cs^+^ in this reaction effectively stabilizes the layered δ-MnO_2_ structure. Morphologies of the samples are shown in Fig. 1b. While all samples contain particles with a diameter of few micrometers, the differences in the surface of the particles are obvious. Combining with XRD patterns, it can be concluded that the urchin-like particles (Fig. 1b) formed after 0.5 and 1 hour reaction time are mainly Mn_2_O_3_ and α-MnO_2_ while the flower-like particles are purely δ-MnO_2_.

**Fig. 2:**
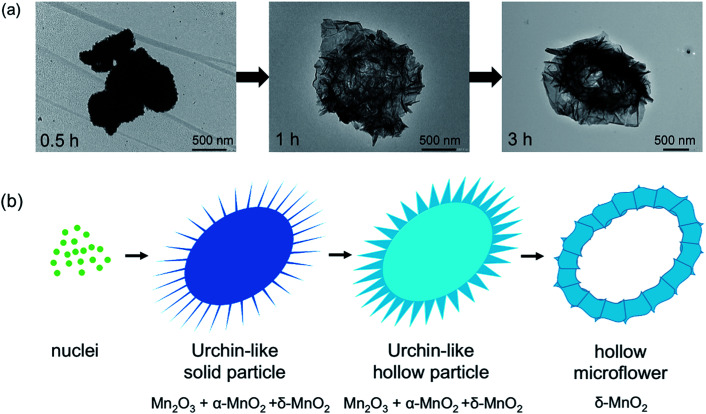
TEM images (a), and formation process (b) of the products from molten salt synthesis. δ-MnO_2_ formation occurs through formation of Mn_2_O_3_ and α-MnO_2_, respectively. Simultaneously, the morphology changes from solid urchin-like particles to hollow microflowers as explained by Ostwald ripening process.

To further explore the morphologies, the samples were investigated by TEM (Fig. 2a), which shows the sample transformation from solid particles to the hollow ones. Based on XRD, SEM, and TEM, formation of the δ-MnO_2_ hollow microflowers could be explained by the ‘Ostwald ripening process’^18^ as illustrated in Fig. 2b. First, a large number of nuclei are formed and later grown into urchin-like solid particles with needle-like surface. At this stage, the sample contains both Mn_2_O_3_ and α-MnO_2_. After 1 hour, sides of the needles grow and the sample transforms to δ-MnO_2_. This transformation completes in 3 hours and the needles continue to grow until they fuse together forming a petal-like network. During this, the inner part of the particles dissolves, diffuses, and recrystallizes at the outer region in a ripening process resulting in a hollow structure.^19^ Interestingly, a similar but reverse formation process was reported in hydrothermal syntheses, where δ-MnO_2_ solid spheres were formed first and later transformed to α-MnO_2_ hollow spheres.^20,21^ This further demonstrates the crucial role of Cs^+^ in stabilizing δ-MnO_2_ phase.

**Fig. 3:**
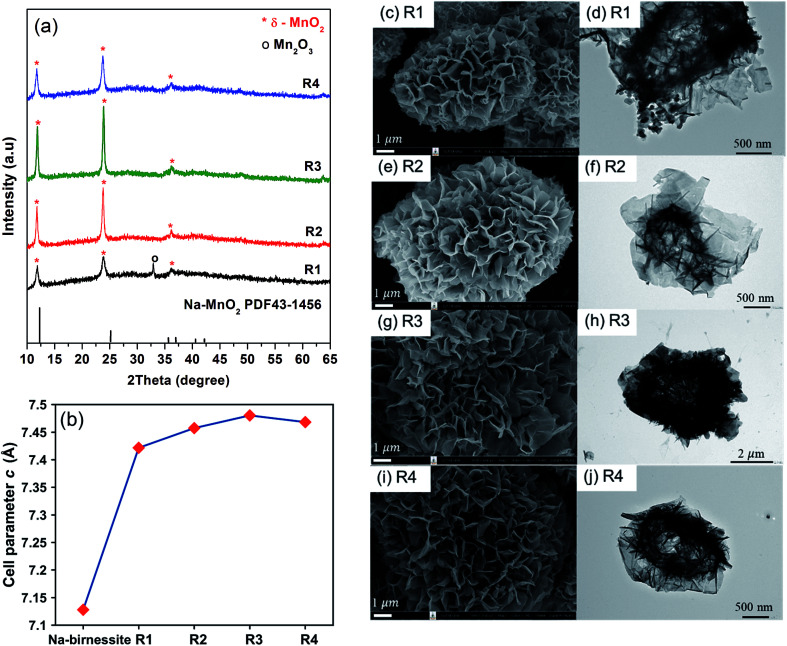
XRD patterns (a) cell parameters, (b) SEM and TEM images, and (c–j) of Cs–MnO_2_ obtained from molten salt synthesis with CsNO_3_ : MnSO_4_ weight ratios of 3 : 1 (R1), 5 : 1 (R2), 7 : 1 (R3), and 10 : 1 (R4) at 430 °C for 3 hours.

The effects of CsNO_3_ : MnSO_4_ ratios are investigated by varying the weight ratio to 3 : 1 (R1), 5 : 1 (R2), 7 : 1 (R3), and 10 : 1 (R4). Stoichiometrically, 3 : 1 salt to reagent weight ratio is required to completely oxidize Mn^2+^ to Mn^4+^. Using exactly this ratio was not enough to get pure MnO_2_, but all reactions using higher ratios gave single-phase δ-MnO_2_ (Fig. 3a). It is noteworthy that the amount of salts does not affect the resulting crystal structures. However, XRD peak intensity indicates that R3 has the highest crystallinity. One possible explanation is that the molten salt acts as a solvent during the reaction and a certain additional amount is required for particle growth, but excessive salts could lead to a dilute system where particle growth is suppressed.

The interlayer distances in our samples (*d*_001_) were calculated from the XRD patterns collected with the internal standard. The obtained values are plotted as a function of salt content in Fig. 3b. For comparison, we prepared Na-birnessite according to a previous report^22^ and similarly calculated the *d*_001_. The *d*_001_ of our samples are comparable to the previous reports of Cs-birnessite and are larger than that of Na-birnessite because of the larger intercalating Cs^+^.^23,24^ Additionally, the relative intensity of (001) at 12° and (002) at 25° in the XRD patterns is different from that of Na-birnessite. The observed relative intensity here suggests the presence of heavy species (Cs^+^) in between MnO_2_ layers.^25^ EDS results (ESI, Fig. S1–S4†) indicate that Cs, Mn, and O are homogeneously distributed in the samples as expected. Based on ICP analyses, Cs to Mn mole ratio is calculated and summarized in Table 1. This ratio appears to changes slightly with the starting salt content. A similar trend is observed in the interlayer distances confirming the Cs^+^ intercalation. However, the interlayer distances saturate at a certain value. Interestingly, Chitrakar *et al.*^24^ studied Cs^+^ ion exchange in Na-birnessite and found that the maximum uptake of Cs^+^ was approximately 2 mmol g^−1^, which would results in a Cs : Mn ratio of 0.17 : 1. The Cs : Mn ratio in our samples are quite similar. Nevertheless, our reactions use much higher Cs^+^ content and temperature, which might be the reason for a slightly higher Cs capacity.

**Table 1:** Elemental compositions, BET surface areas, and pore volumes of the samples

Sample	CsNO_3_ : MnSO_4_ weight ratios	Cs : Mn mole ratio in the sample	Surface area (m^2^ g^−1^)	Pore volume (cm^3^ g^−1^)
R1	3 : 1	0.17	39.5	0.27
R2	5 : 1	0.19	34.5	0.21
R3	7 : 1	0.20	29.2	0.15
R4	10 : 1	0.24	47.6	0.27

Based on the SEM images (Fig. 3c–j), the general features of the microflowers are unaffected by the salt content. The only observed difference is the thickness of the petal parts which seems to become thinner with increasing salt content. Although the salt to reagent ratio influences the crystallinity as previously discussed, it has little to no effects on BET surface areas and pore volumes of the samples (Table 1). The adsorption/desorption isotherm and pore size distribution of the samples are shown in ESI, Fig. S5–S8.†

**Fig. 4:**
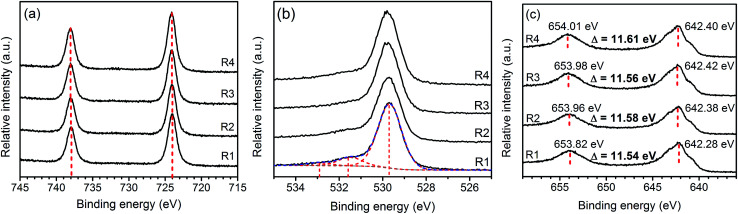
XPS spectra: Cs3d (a), O2p (b), and Mn2p (c) of Cs–MnO_2_ obtained from molten salt synthesis with CsNO_3_ : MnSO_4_ weight ratios of 3 : 1 (R1), 5 : 1 (R2), 7 : 1 (R3), and 10 : 1 (R4).

Fig. 4 presents Cs3d, O2p, and Mn2p XPS spectra of the samples. The Cs3d_5/2_ and 3d_3/2_ XPS spectra at 724 and 738 eV are the characteristics of Cs^+^.^26^ The O1s peak can be deconvoluted into three components including the lattice oxygen (∼530 eV), oxygen from contamination during sample preparation (∼532 eV), and absorbed species (∼533 eV).^27^ Both the positions and separations of Mn2p_3/2_ and Mn2p_1/2_ peaks suggest that oxidation state of Mn in the samples are in between that of MnO_2_ and Mn_3_O_4_. Similar results were observed in other ion-intercalated MnO_2_ birnessite as the partial reduction of Mn(iv) is necessary to accommodate the intercalating ions.^16^

### 3.2. Electrochemical properties

**Fig. 5:**
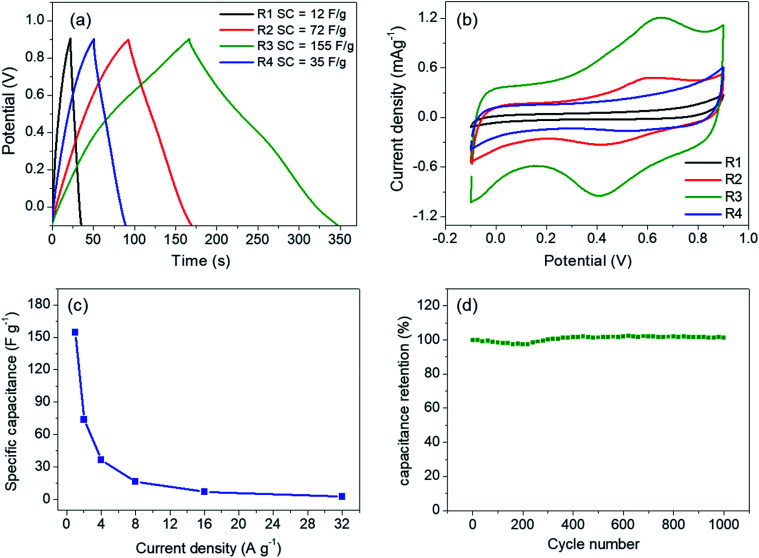
(a) GCD curves at 1 A g^−1^ and cyclic voltammograms at 5 mV s^−1^ (b) of the samples obtained from molten salt synthesis with CsNO_3_ : MnSO_4_ weight ratios of 3 : 1 (R1), 5 : 1 (R2), 7 : 1 (R3), and 10 : 1 (R4). (c) and (d) show rate capability and cyclic performance at 5 A g^−1^ of R3, respectively.

The linear and symmetric GCD curves of the samples (Fig. 5a) demonstrate good capacitive behavior.^17^ The specific capacitances of the samples are in between 12–155 F g^−1^ at current density of 1 A g^−1^. Despite the similar physical properties, R3 shows the highest specific capacitance. To rationalize such a difference, the prepared electrodes were investigated by cyclic voltammetry (Fig. 5b). The capacitive behaviors in MnO_2_ are based on two mechanisms. The electric double layer capacitance (EDLC) relies on the adsorption/desorption of electrolytic ions at the electrode surface while the pseudocapacitance occurs through redox reactions of the electrode materials. The first mechanism depends mostly on the surface area while the latter is promoted by ion intercalation/deintercalation.^13^ In our case, the low surface areas and the low rate capability (Fig. 5c) suggest that the contribution from pseudocapacitance is dominant, especially in R3 sample, which shows clear redox peaks at 0.42 and 0.65 V.^14,28^ In fact, a similar redox peak observed in K-intercalated MnO_2_ had been attributed to faradaic ion deintercalation of the electrolytic ions.^13^ Such the intercalation/deintercalation is promoted by the expanded interlayer distance. Thus, the capacitance is expected to increase with *d*_001_.^28^ Therefore, the pronounced redox activity of R3 is attributed to the high crystallinity and the optimum amount of Cs^+^, which maximizes the interlayer distance. On the other hand, R4 sample contains higher Cs content but has a slightly lower interlayer distance, which do not promote intercalation/deintercalation process as evidenced from a much lower specific capacitance. It should be noted that our Cs–MnO_2_ (R3) exhibits similar specific capacitance to those of Li-, Na-, and K-intercalated MnO_2_ (140–160 F g^−1^), which has comparable surface area (50–70 m^2^ g^−1^).^15^ Additional comparison of the specific capacitance of Cs–MnO_2_ with other alkaline-intercalated MnO_2_ is summarized in Table 2. Additionally, the electrode prepared from R3 shows good cycle stability by maintaining nearly 100% of its capacitance after 1000 cycles (Fig. 5d). It is known that the specific capacitance and electrochemical behaviors of pre-intercalated MnO_2_ could be further improved by increasing the surface area or compositing with other functional materials.^13,14,16^ Thus, this work has shown that Cs–MnO_2_, in addition to other alkaline-MnO_2_, could be an interesting candidate for supercapacitor electrodes.

**Table 2:** The specific capacitance of Cs–MnO_2_ compared with other alkaline-intercalated MnO_2_

Materials	Morphology	BET surface area	Electrolyte	Loading mass (mg cm^−2^)	Specific capacitance (F g^−1^)	Cycle stability of electrode	References
Cs_0.2_MnO_2_	Microflower	29 m^2^ g^−1^	0.5 M K_2_SO_4_	1	155 at 1 A g^−1^	∼100% after 1000 cycles	This work
Li^+^-layered MnO_2_	Nanosheet	70 m^2^ g^−1^	0.2 M Li_2_SO_4_	N/A	147 at 0.5 mA cm^−2^	99% after 1000 cycles	29
Na^+^-layered MnO_2_	Nanosheet	53 m^2^ g^−1^	0.2 M Li_2_SO_4_	N/A	163 at 0.5 mA cm^−2^	94% after 1000 cycles	29
Na^+^-layered MnO_2_	Nanoflake	N/A	1 M Na_2_SO_4_	0.03–2.25	∼155 at 2.2 A g^−1^	99.9% after 1000 cycles	28
K^+^-layered MnO_2_	Nanosheet	50 m^2^ g^−1^	0.2 M Li_2_SO_4_	N/A	139 at 0.5 mA cm^−2^	94% after 1000 cycles	29
K–MnO_2_	Nanobelts	N/A	0.5 M K_2_SO_4_	3–5 mg	130 at 1 A g^−1^	100% after 100 cycles	30
K_0.15_MnO_2_·0.43H_2_O	Nanosheet	N/A	0.1 M Na_2_SO_4_	3.8 × 10^−2^	303 at 0.2 A g^−1^	N/A	31
K_0.6_MnO_2_	Nanosheet	N/A	1 M potassium bis(trifluoromethanesulfonyl)-imide	N/A	254 at 1 A g^−1^	N/A	32

## 4. Conclusions

Molten salt synthesis employing CsNO_3_ is successfully used to prepare Cs–MnO_2_ hollow microflowers with layered structure. Formation of δ-MnO_2_ occurs through several stages including the formation of Mn_2_O_3_ and α-MnO_2_. Simultaneously, the morphology transforms from solid urchin-like particles to hollow microflowers, which is explained based on the Ostwald ripening process. The expanded interlayer distance, the XPS spectra, and the elemental analysis indicate that the samples are Cs-intercalated δ-MnO_2_. Investigation on the effects of salt to reagent ratio suggests that the ratio has little effects on the crystal structure and morphologies. However, both the interlayer distance and the crystallinity change with the salt content. The sample obtained from 7 : 1 salt to reagent ratio shows the best specific capacitance of 155 F g^−1^ and a good cycling stability due to its suitable crystallinity and interlayer distance.

## Conflicts of interest

There are no conflicts to declare.

## Supplementary Material

RA-009-C9RA02067E-s001
