# Supplementary material for: Facile molten salt synthesis of Cs–MnO2 hollow microflowers for supercapacitor applications
Source: RSC Adv. 2019 Jun 17;9(33):19079–85. doi: 10.1039/c9ra02067e (PMC9065054; doi:10.1039/c9ra02067e)
Supplement: RA-009-C9RA02067E-s001 [file RA-009-C9RA02067E-s001.pdf]

Electronic Supplementary Material (ESI) for RSC Advances.
This journal is © The Royal Society of Chemistry 2019

Supporting Information

Facile molten salt synthesis of Cs-MnO_2 hollow microflowers for supercapacitor applications

Praeploy Chomkhuntod[a], Arreerat Jiamprasertboon[a], Anurak Waehayee[a], Teera Butburee[b], Narong Chanlek[c], Nararat Yong[a], Theeranun Siritanon*[ad]

[a]School of Chemistry, Institute of Science, Suranaree University of Technology, Nakhon Ratchasima, Thailand

[b]National Nanotechnology Center, National Science and Technology Development Agency, 111 Thailand Science Park, Pathum Thani, Thailand

[c]Synchrontron Light Research Institute, Nakhon Ratchasima, Thailand.

[d]Center of Excellent-Advanced Functional Materials, Suranaree University of Technology, Nakhon Ratchasima, Thailand

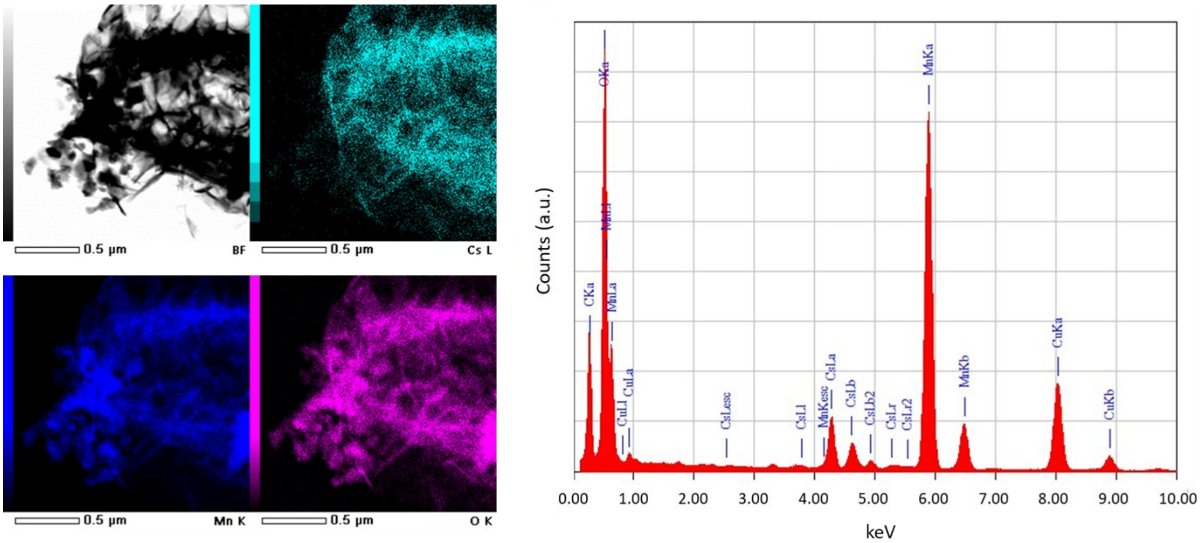

Fig. S1. EDS mapping and EDS spectrum of sample R1

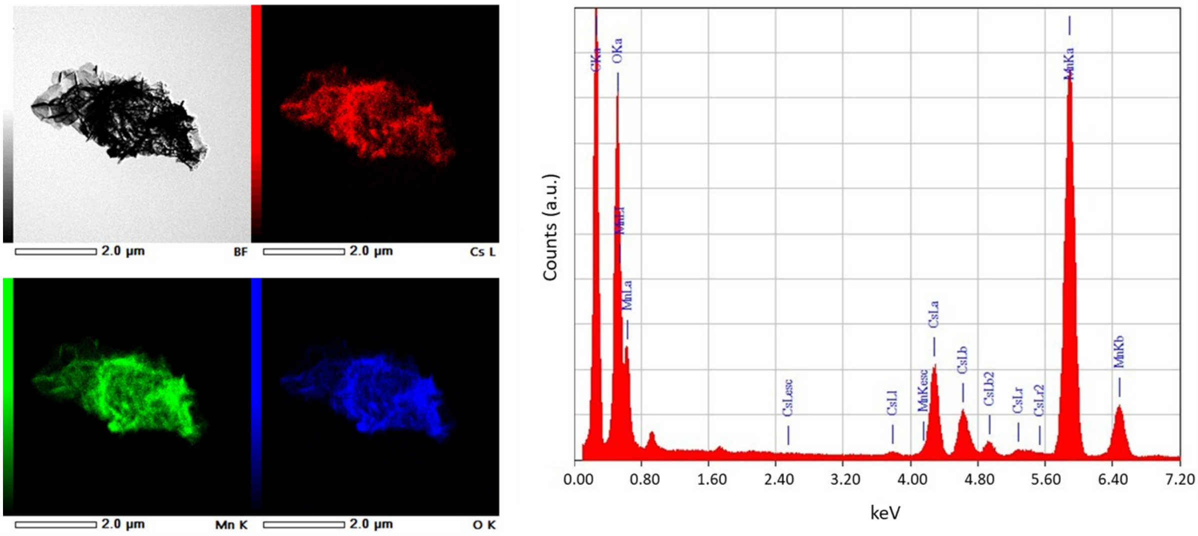

Fig. S2. EDS mapping and EDS spectrum of sample R2

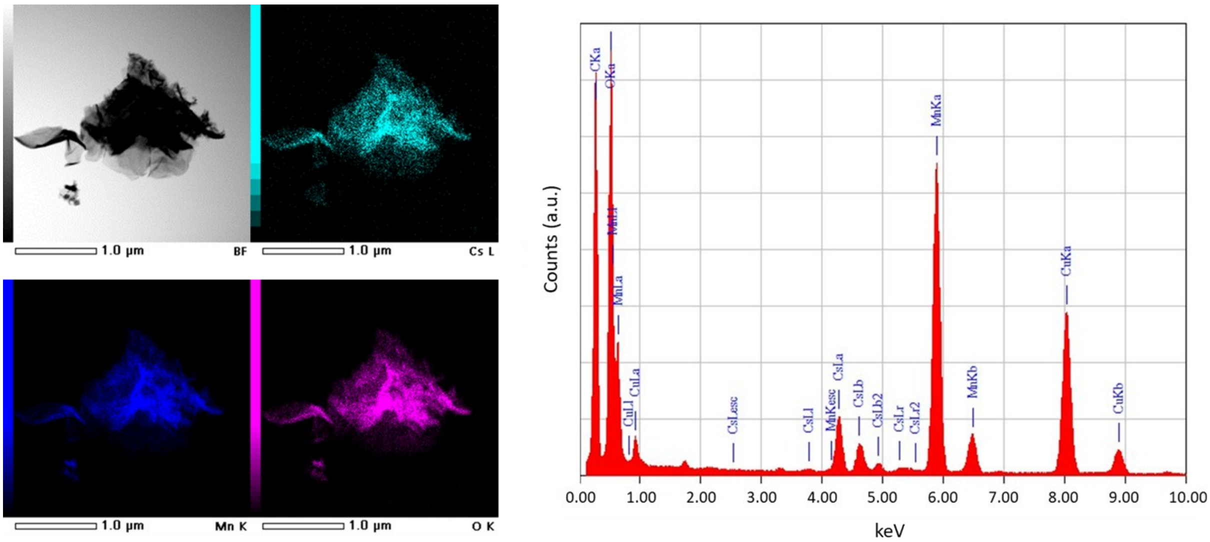

Fig. S3. EDS mapping and EDS spectrum of sample R3

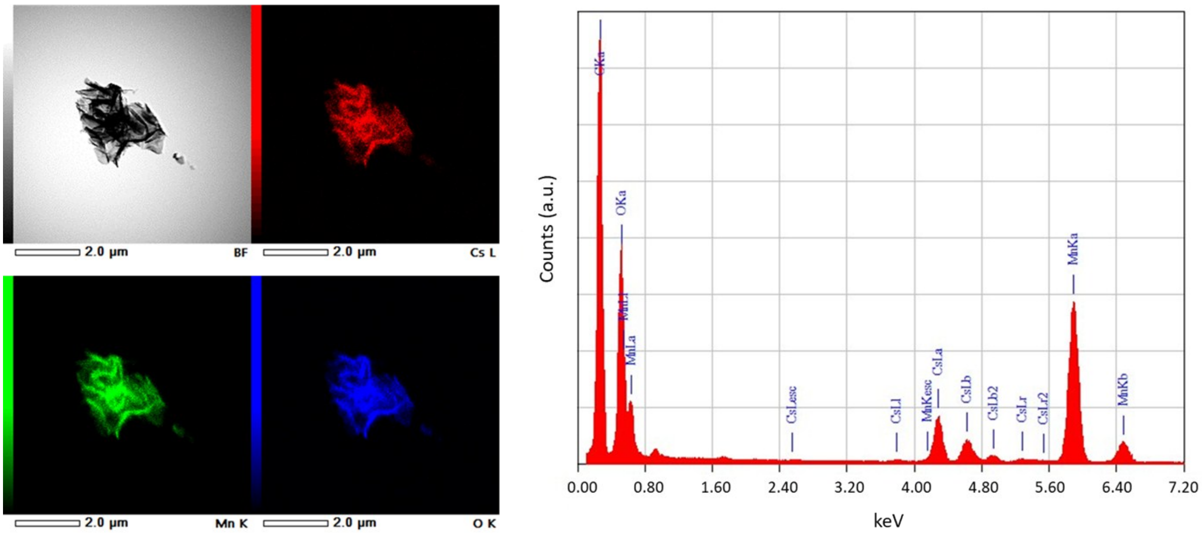

Fig. S4. EDS mapping and EDS spectrum of sample R4

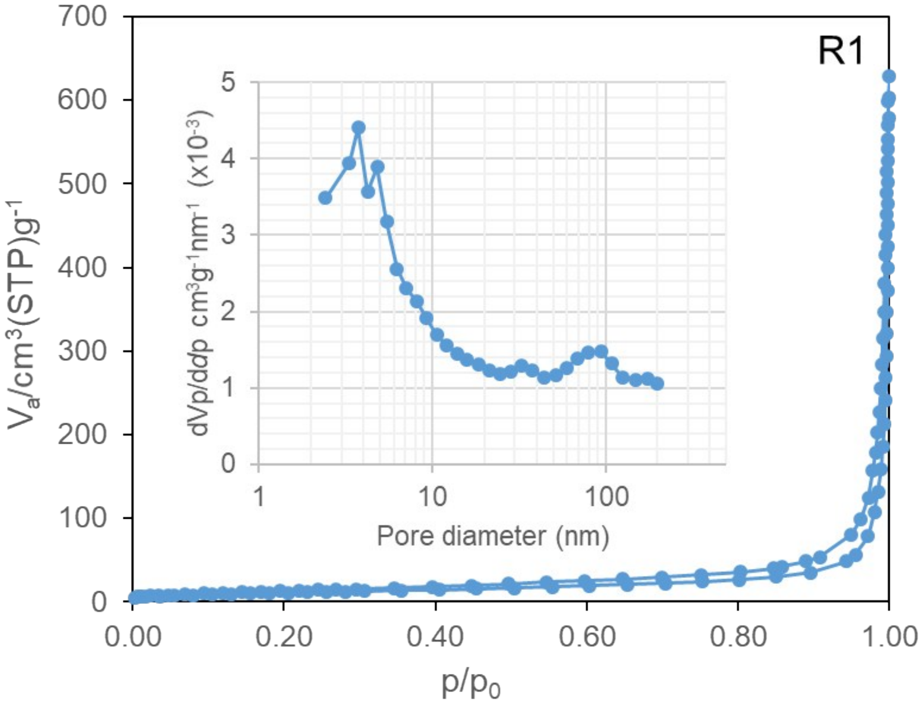

Fig. S5. Adsorption/desorption isotherm and pore size distribution of sample R1

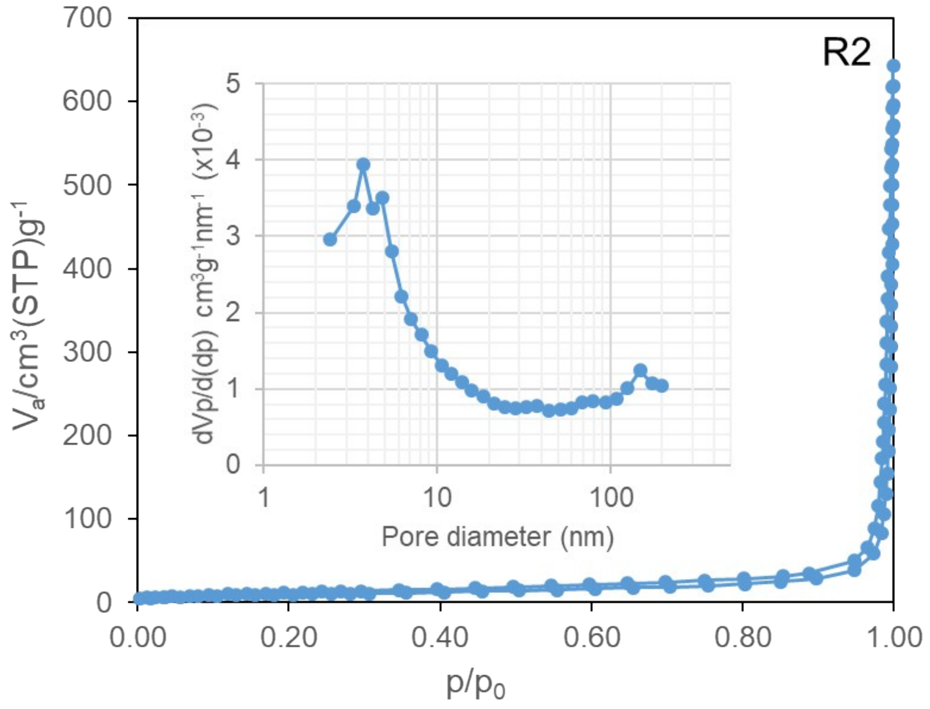

Fig. S6. Adsorption/desorption isotherm and pore size distribution of sample R2

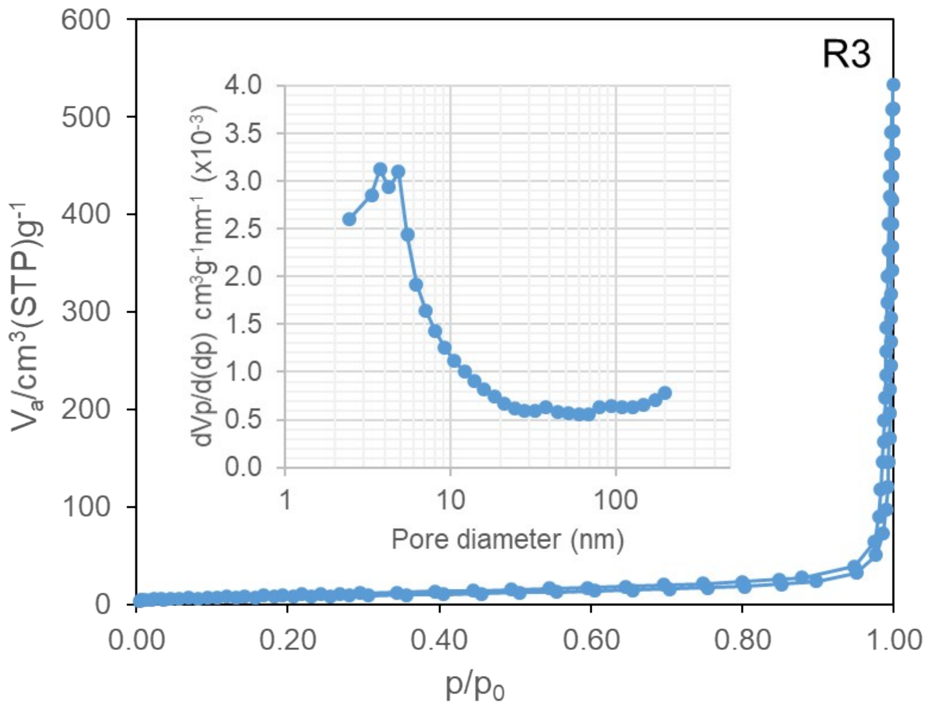

Fig. S7. Adsorption/desorption isotherm and pore size distribution of sample R3

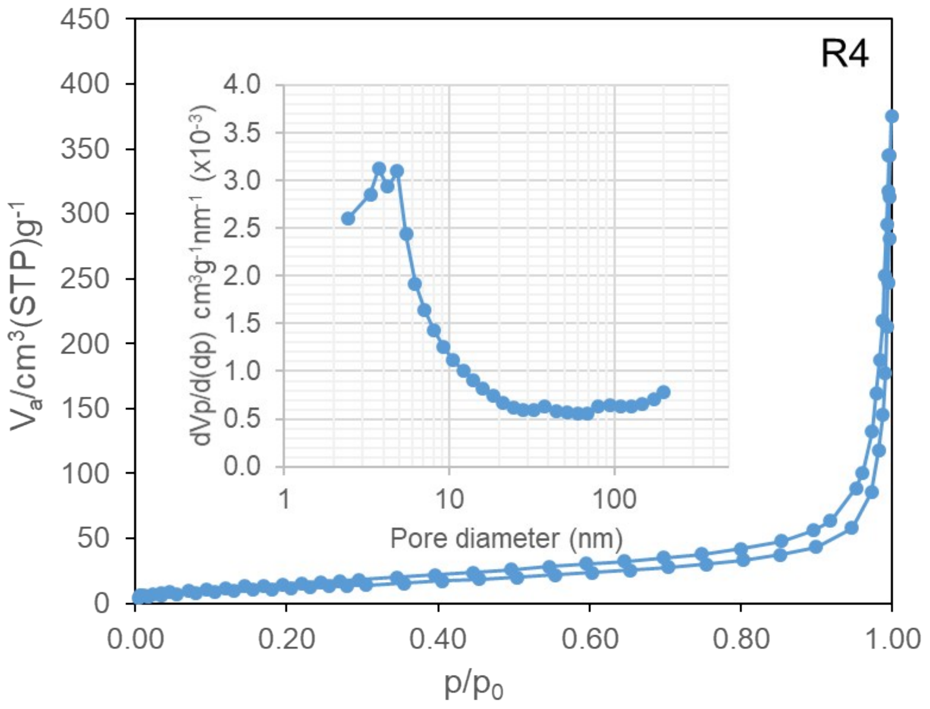

Fig. S8. Adsorption/desorption isotherm and pore size distribution of sample R4
